# Organizational climate of kindergartens and teacher professional learning: mediating effect of teachers’ collective efficacy and moderating effect of mindfulness in teaching

**DOI:** 10.3389/fpsyg.2024.1287703

**Published:** 2024-04-09

**Authors:** Shuang Hao, Dongqing Yu, Lisha Fu

**Affiliations:** Faculty of Education, Northeast Normal University, Changchun, China

**Keywords:** kindergarten, organizational climate, teacher professional learning, collective efficacy, mindfulness in teaching, moderated mediation

## Abstract

**Introduction:**

This study was aimed at testing a moderated mediation model of teaching mindfulness and teachers’ collective efficacy in the relationships between the organizational climate of kindergartens and teacher professional learning.

**Methods:**

A sample of 1,095 kindergarten teachers completed self-report questionnaires assessing their perceptions of the organizational climate of kindergartens, collective efficacy, teaching mindfulness, and professional learning.

**Results:**

Controlling for teaching experience and kindergarten level, the results show that kindergarten organizational climate significantly and positively predicted teacher professional learning and the collective efficacy of teachers played a partial mediating role between them.

Furthermore, moderation analysis revealed that teaching mindfulness moderated the relationship between kindergarten organizational climate and teacher professional learning.

**Discussion:**

These results expand our understanding of how the organizational climate of kindergartens affects teacher professional learning. In practice, professional learning of kindergarten teachers can be facilitated by creating an open organizational climate and improving their ability to perceive the collective. Furthermore, the moderating role of teaching mindfulness suggests that intervening in teachers’ teaching mindfulness possibly is an influential way to maximize the impact of kindergarten organizational climate on professional learning.

## 1 Introduction

Teacher professional learning (TPL) is increasingly in the spotlight as an important means and modality of teacher professional development, and it is considered one of the most significant forecasters of school improvement, optimization of instructional practices (Thoonen et al., 2011), and improved student learning (Kelly, 2006; Doğan and Yurtseven, 2018). Sustained learning opportunities often occur during job-embedded activities where teachers exchange ideas and share knowledge. Thus, TPL is seen to occur in both teachers’ personal and professional actions and mutually beneficial social interactions (Gibbons and Cobb, 2017). Based on this, we define TPL as the mix of individual and collaborative teacher activities to build pedagogical knowledge and develop professional skills for teaching in a particular learning system (Bakkenes et al., 2010). A set of experimental data suggests that specific professional learning activities contribute significantly to teachers’ efficacy and that teachers’ seniority did not influence this path (Sulla and Rollo, 2023). Thus, it seems that the learning and development of kindergarten teachers is an important part of improving the quality of ECE education (Schachter et al., 2019). The Chinese government attaches particular importance to the professional development of teachers and has established a five-tier training system at the national, provincial, municipal, county, and school levels (Ministry of Education of the People’s Republic of China, 2021a, 2021b). Over the past 10 years, the Chinese government has implemented the National Training Program for Kindergarten Teachers (NTPKT), which has trained 2.43 million kindergarten teachers from 2012 to 2020, and has resulted in a significant improvement in the professionalism of teachers (Ministry of Education of the People’s Republic of China, 2022). The program continues to advance in 2021, with a new five-year training program having been launched (Ministry of Education of the People’s Republic of China, 2021a, 2021b). A series of supportive initiatives by the Chinese government have had a positive and wide-ranging impact, contributing to the improvement of the quality of preschool education in all regions of China. TPL is a complex task because it requires sustained commitment and effort and is often extremely challenging for individuals (Cooper et al., 2020). This perspective highlights the school as a learning setting for both teachers and students. Many studies suggest that teachers’ participation in professional learning activities may be linked to school support (Greenglass et al., 1997), and that intentional support provided by schools can consciously motivate teachers to participate in professional learning initiatives (Leithwood et al., 1999). In the kindergarten setting in particular, TPL is heavily influenced by the teacher’s practice setting, as regular kindergarten instruction and caregiving tasks often take up a great deal of time, with limited time left for teacher learning (Cherrington and Thornton, 2015). The support and improvement from the school environment can give chances for kindergarten teachers to grow and improve their educational performance, making the school a place for teacher learning (Kwakman, 2003).

As a relatively stable and lasting characteristic of the school environment (Kottkamp et al., 1987), the organizational climate (OC) provides teachers with a cultural resource, which is reflected mainly in the quality of interpersonal relationships among school staff (Rudasill et al., 2018) (such as the relationship among colleagues, teachers, and students, and the relationship among principals), in which leaders play a major role (Sanchez et al., 2022). Evidence from diverse cultural backgrounds in Asian societies confirms the impact of principal leadership on TPL (Hairon and Dimmock, 2012; Qian and Walker, 2013; Somprach et al., 2017) and suggests that leadership practices may directly affect teacher learning and that, in addition to principal leadership, the support that teachers receive from their colleagues is closely related to their motivation for professional learning (Ishler et al., 1998; Zhang et al., 2021). All these factors affecting TPL are related to interpersonal interaction and relationship quality within the school. However, to date there have been only a few studies on the relationship between OC with overall relationship quality as the core and TPL. To fill this gap, we explore the intentional support implicit in the school environment (i.e., OC) that affects TPL by examining the roles of collective efficacy (CE) as well as mindfulness in teaching.

## 2 Literature review and hypotheses

### 2.1 Organizational climate and teacher professional learning

As a characteristic presented by the school environment, OC can be defined by the relationships among members of the organization and by teachers’ shared perceptions of behavior (Hoy and Tarter, 1992). This can affect teachers’ cognitive development, behavioral performance, and personal feelings (Skaalvik and Skaalvik, 2009; Collie et al., 2012; Malinen and Savolainen, 2016). It has been noted that school culture, climate, and the learning environment experienced by teachers can all influence TPL and thus improve their teaching practices (Kwakman, 2003; Furner and McCulla, 2019; Nguyen et al., 2021; McChesney and Cross, 2022). Organizational support has a significant role in influencing employees’ engagement in their work (Bonaiuto et al., 2022). Among these, principal support can provide the resources, systems, and structures that facilitate TPL and set the tone of the OC, particularly the climate of trust, and teachers’ trust in their principals positively influences their professional learning (Bryk et al., 2010; Huang et al., 2021; Bektaş et al., 2022). Liu et al. (2016) also found that teachers’ trust in colleagues can positively influence collaboration, reflection, and innovation in TPL, and this result has also been verified in kindergarten teachers (Yin et al., 2019; Karacabey et al., 2022).

The model proposed by McMillan et al. (2016) identifies interpersonal relationships as one of the key influences on teachers’ perceptions and motivation for engaging in continuing professional development. Establishing and maintaining these harmonious and trusting relationships facilitates an open and supportive OC, which leads to more-active teacher participation in professional learning (Liu et al., 2014). Hoy (2023) noted that school leaders and teachers have different patterns of behavior and that school climate can be characterized by varying degrees of openness or closedness depending on the level of openness of leader and teacher behavior. In particular, the openness of principals influences teachers’ attitudes toward learning and enhances their willingness to learn (Park et al., 2019). That is, if teachers receive more organizational support in the process of professional learning (positive OC), They would be more likely to attend professional learning programs in kindergarten. On the contrary, kindergarten teachers in a closed and negative climate will have a lower willingness to engage in professional learning, which is not favorable to the efficacy of professional learning. Therefore, on the basis of the above, we make the hypothesis below.

*H1*: Kindergarten organizational climate has a significant positive effect on teacher professional learning.

### 2.2 Mediating role of collective efficacy

As a collective motivator, CE depicts teachers’ perspectives on their collective capacity to utilize their own resources to meet difficulties or challenges and to generate and enrich successful learning contexts (Hoy and Miskel, 1978; Goddard et al., 2000), and it is derived from group characteristics that are reflected as a result of inter-individual interactions (Bandura, 1997; Meyer et al., 2022). This process of the environment acting on individual learning is affected by motivation, interest, beliefs, and other internal factors (Er, 2021). Teachers’ CE and willingness to make changes are affected by the resources of the environment in which they work (Daly, 2010). This workplace collective culture is referred to as “climate,” “informal organization,” or “culture.” Thus, CE plays an important role between the external environmental dimension of OC and the individual behavior of TPL.

Related research confirms that OC is directly related to CE. A positive OC promotes more-active participation in work and willingness to help colleagues to solve problems together by enhancing communication feedback from school members (Castro Silva et al., 2017), thus increasing teachers’ confidence in their collective competence (Loughland and Ryan, 2022). Based on Bandura’s social cognitive theory, efficacy is engendered by the social experiences that provide the basic information that allows individuals to form opinions about their ability to execute some actions in order to achieve the desired results (Bandura, 1997). Leader behaviors and teacher behaviors in the school climate are the social sources of teachers’ CE. Effective school leadership behavior is a positive and important predictor of teachers’ CE perceptions (Cansoy and Parlar, 2018; Ma and Marion, 2021). It has been argued that principals play the most determining role in the formation of the school climate (Leithwood et al., 2010) and that principals’ leadership behaviors can influence CE by increasing the opportunities for teachers to work collaboratively around improving teaching and learning (Goddard et al., 2017; Versland and Erickson, 2017; Goddard et al., 2021). Besides leadership behaviors, teachers’ interactions with colleagues are equally important. Conflict/trust among colleagues was included as a predictor of the social or organizational dimensions affecting teacher efficacy effectiveness and was significantly related to teachers’ CE (Goddard et al., 2015). A increasing amount of research suggests that teachers have higher CE in schools where they work collaboratively, believe in common objectives, and experience cooperation (Mackenzie, 2000; Collie et al., 2012; Goddard et al., 2015; Voelkel Jr. and Chrispeels, 2017; Loughland and Ryan, 2022). Teachers are a collective group, not merely individual educators in a school, and will unavoidably be affected by the context in which they work (Collie et al., 2012). It follows that creating a school climate that is centered on promoting teacher learning can enhance teachers’ CE.

As one of the efficacy belief structures, CE can have an effect on individual learning (Bandura, 1997). Opfer and Pedder (2011) found that an important factor influencing TPL was school-level beliefs about learning. Teachers with a high level of CE are resilient in terms of surmounting obstacles to educating their students. Teachers embrace challenges, set targets to meet them, and have a strong belief in collective competence as well as a focus on how collectively they can work together effectively, thus furthering their attention to student acquisition and growth (Goddard et al., 2004; Karacabey et al., 2022). Related research also indicates that teachers’ CE shapes the values they place on conducting in-depth learning, their attitudes, and patterns of their participation in specialized professional learning activities (Liu and Hallinger, 2018; Hosseingholizadeh et al., 2020). Donohoo (2017) found through his review that professional learning occurs in cooperative and not insular environments, and all require the need to utilize the power of the collective. Therefore, teachers who are more CE-aware are more likely to be actively involved in professional learning activities. In summary, a positive kindergarten climate will promote higher levels of teachers’ CE, which in turn will influence their professional learning. Thus, we propose the following hypothesis.

*H2*: Teachers’ collective efficacy plays a mediating role in the organizational climate of kindergartens and teacher professional learning.

### 2.3 Moderating effect of mindfulness in teaching

Mindfulness is considered an internal individual psychological resource, referring to an individual’s conscious and nonjudgmental attention to internal and external stimuli, such as physical sensations, emotional reactions, and thinking in the present moment (Bishop et al., 2004). This conceptualization places more emphasis on intra-individual states but makes less mention of the interpersonal dimension of mindfulness. Frank et al. (2016) proposed studying mindfulness in teaching within the context of teaching behaviors, where mindfulness in teaching is assessed in terms of both intrapersonal and interpersonal mindfulness in teaching. Based on the individual–environment interaction model, the environment and the individual interact with each other to influence the development of individual behavior (Lerner et al., 2006). There may be individual differences in the mechanisms by which OC affects TPL because individuals’ conditions vary and the same environment affects different individuals differently. Research has shown that teachers with high positive thinking have stronger regulatory abilities and that teachers can consciously remind themselves of the current environmental situation and adjust their behavior in time through mindfulness (Roeser et al., 2022). Insight into teachers’ psychological mechanisms is important in explaining the relationship between the work environment and TPL (De Wal et al., 2020). Therefore, it is necessary to introduce an individual-trait variable (i.e., mindfulness in teaching) to explore the moderating role of individual traits in the above model.

Resource conservation theory points out that resources have an initial resource effect that reduces the loss of resources and increases the gain of new resources (Hobfoll et al., 2018). Being a positive individual trait, mindfulness has a positive impact not only on an individual’s cognitive functioning, emotional regulation, and behavioral adaptation but is also an important protective factor for an individual’s developmental adaptation (Kalafatoğlu and Turgut, 2019). In the workplace, people who have high levels of mindfulness are much more likely to adapt to their environment and have greater resilience, job engagement, job contentment, and well-being (Song et al., 2021; Tao, 2022). When individuals have high levels of mindfulness, focused self-awareness and concentration may help them to clearly see results, increasing their readiness to strive for self-improvement and autonomy in their roles, as well as helping them to realize that in the wider work context, they will be far less prone to miss significant inner states or outer clues that can inform their learning (Schultz et al., 2015). At present, mindfulness is gradually being incorporated into early protection and education, and mindfulness can be used as an internal mechanism to improve an individual’s relationship with perceived well-being (Pan et al., 2022) and stress (Cheng et al., 2020). Mindfulness in teaching is a unique way to assess teachers’ performance of mindfulness in interpersonal and interpersonal behavior (Frank et al., 2016), but the mechanism of action in ECEC (early childhood education and care) educators remains to be explained (Seo and Yuh, 2022). At the same time, mindfulness in teaching is usually used to alleviate negative work states (e.g., stress, burnout, etc.) and lacks validation of the positive transformative effect on objective environmental conditions. In fact, the role of teachers’ mind’fulness in teaching in the educational environment is crucial for the self-adjustment and transformation of existing resources, in addition to alleviating negative states. Therefore, this study was focused on examining how mindfulness in teaching transforms the objective environmental condition of OC and thus influences TPL. Accordingly, we put forward the following hypothesis.

*H3*: Teachers’ mindfulness in teaching plays a moderating role in the influence of organizational climate on teacher professional learning.

**Figure 1:**
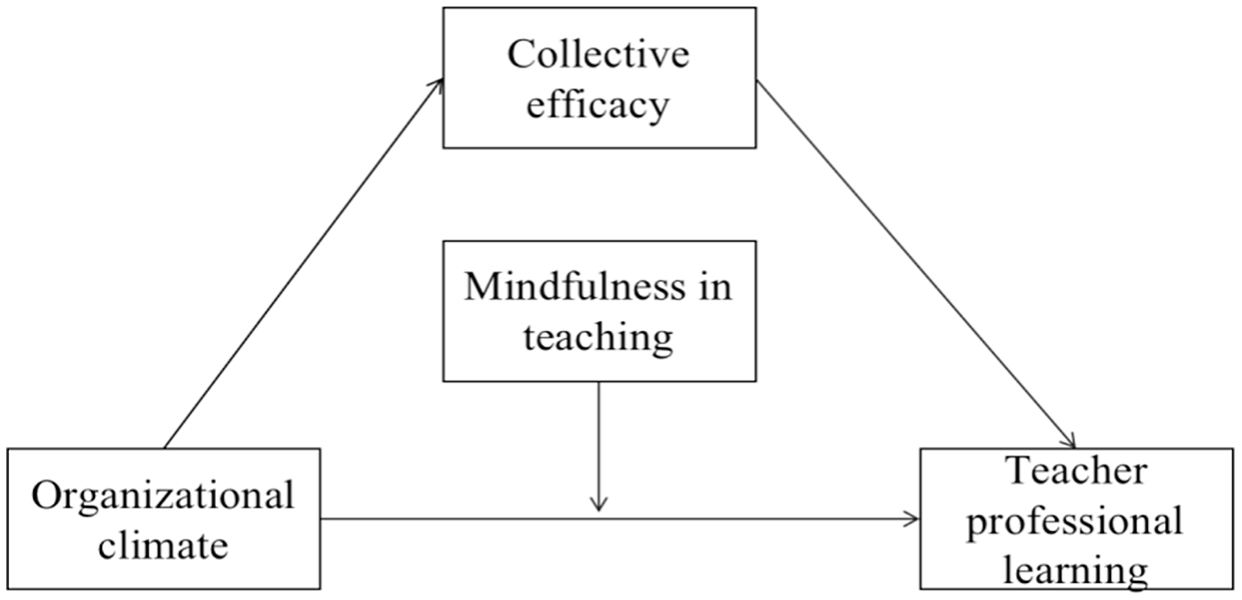
Proposed theoretical model.

In this context, we design a moderated mediation model (Figure 1) that provides a clear picture of how OC impacts TPL by teacher CE and mindfulness in teaching.

## 3 Methodology

### 3.1 Participants and data collection

In this study, the convenience sampling method was used to recruit early childhood teachers from different regions of Northeast, North, Central, Southwest, and Northwest China from March to May 2023 to complete the questionnaire. The questionnaires were forwarded to the kindergarten teachers through the principals and collected through the online survey platform “Questionnaire Star.” The survey took approximately 15 min to complete. At the start of the survey, informed consent was obtained, and all participating teachers were informed that their participation was strictly voluntary. After excluding questionnaires that were completed too quickly (<3 min) and incomplete responses, we obtained 1,095 valid questionnaires. Their teaching experience ranged from zero to 20 years. The specific demographic information of the sample is given in Table 1, comprising teachers’ seniority and educational background, as well as the level, type, and position of kindergarten.

**Table 1:** Participant characteristics (*N* = 1,095).

Demographic characteristic		*N*	%
Seniority	5 years or less	424	38.7
	6–10 years	290	26.5
	11–15 years	151	13.8
	16 years or more	230	21.0
Educational background	Junior college or lower	417	38.1
	Bachelor degree	661	60.4
	Master’s degree or above	17	1.6
Level of kindergarten^a^	Demonstration	810	74.0
	First-level	126	11.5
	Second-level or lower	159	14.5
Type of kindergarten	Public	881	80.5
	Private	214	19.6
Position of kindergarten	City	644	58.8
	County	243	22.2
	Countryside	208	19.0

a The level of kindergarten is assessed provincially according to a hierarchy from highest to lowest: demonstration kindergarten, first-level kindergarten, second-level kindergarten, and third-level kindergarten.

### 3.2 Measures

#### 3.2.1 Questionnaire on organizational climate

We used the Chinese version of the Kindergarten Organizational Climate Description (OCDQ-RE) questionnaire, which was developed originally by Hoy and Tarter (1992) and adapted by Li et al. (2017) for kindergarten teachers. The questionnaire consisted 33 items in six dimensions to describe teachers’ perceptions of the organizational atmosphere: (i) “Supportive principal behavior (SPB)” (six items) references to support and care for teachers by principals; (ii) “Directive principal behavior (DPB)” (six items) referred to whether the principal’s leadership was assignment-oriented, showed little attention to teachers, and involved close supervision and little delegation of authority to teachers; (iii) “Restrictive principal behavior (RPB)” (four items) referred to the principal’s tendency to ask teachers to do additional work that was not directly related to instruction; (iv) “Collegial teacher behavior (CTB)” (eight items) addressed the level of appreciation among teachers and their readiness to collaborate and engage in discussions about challenges related to teaching and learning; (v) “Intimate teacher behavior (ITB)” (four items) referred to the extent to which teachers care about each other; (vi) “Disengaged teacher behavior (DTB)” (five items) involves teachers’ perception of not belonging to the kindergarten organization, being at a certain remove from co-workers and the organization, and not having common goals for the organization. The Cronbach’s alphas for the six dimensions in Li et al.’s study ranged from 0. 66 ~ 0. 87. Teachers were asked to rate each item on a five-point Likert scale ranging from “strongly disagree” to “strongly agree.” We adopted Hoy’s concept of climate openness (Hoy and Ocdq, n.d.), and indices of the degree of openness in principal–teacher relations were calculated by first normalizing the school scores on these dimensions (with each dimension being a standard score), i.e., principal openness = SPB − DPB − RPB and teacher openness = CTB + ITB − DTB. Then, the kindergarten climate openness index was calculated as KCO = (SPB − DPB − RPB) − (CTB + ITB − DTB) (Barnová et al., 2022). The value of Cronbach’s alpha for this questionnaire was 0.8, Cronbach’s alpha in the range of 0.65–0.88 for each dimension.

#### 3.2.2 Collective teacher efficacy scale

We used the Collective Teacher Efficacy scale created by Tschannen-Moran and Barr (2004). The scale has been validated to be suitable for the Chinese context and has good construct validity and reliability, and the Cronbach’s alpha coefficient was 0.96 (Zhang and Liu, 2023). The scale consisted of 12 self-assessment items in two dimensions, i.e., instructional strategies and student discipline, with teachers asked to assess their beliefs about the collective ability to influence student learning. All questions were scored on a five-point Likert scale ranging from 1 (never) to 5 (always), with higher scores indicating higher levels of teachers’ CE. In this study, the Cronbach’s alpha values for the two subscales of Teaching Strategies and Student Discipline were 0.91 and 0.91, respectively, and the Cronbach’s alpha value for the total scale was 0.95.

#### 3.2.3 Mindfulness in teaching scale (Chinese version)

The Mindfulness in Teaching Scale (MTS) was initially developed by Frank et al. (2016) to assess the mindfulness levels of teachers. We used the Chinese version of MTS (MTS-C) as revised by Ma et al. (2022). This 14-item scale had two dimensions: intrapersonal mindfulness (Cronbach’s alpha = 0.93) and interpersonal mindfulness (Cronbach’s alpha = 0.73). Items were scored on a five-point Likert scale ranging from 1 (never) to 5 (always), where the intrapersonal mindfulness dimension was reverse scored. Higher scores indicate that kindergarten teachers have higher levels of mindfulness in their teaching. MTS-C has been shown to have good effectiveness and reliability (Ma et al., 2021; Yang et al., 2023). In the present study, the Cronbach’s alpha values for the two subscales of teacher intrapersonal mindfulness and teacher interpersonal mindfulness were 0.94 and 0.78, respectively, and the Cronbach’s alpha value for the total scale was 0.75.

#### 3.2.4 Teacher professional learning scale

The eight-item Teacher Professional Learning Scale was extracted from Li et al. (2016). Teachers were asked to grade each item on a five-point Likert scale ranging from “strongly disagree” to “strongly agree.” The scale was validated in the context of Hong Kong, China (Cronbach’s alpha = 0.90), and the results supported the applicability of the scale to kindergarten teachers (Yin et al., 2019). In this study, the value of Cronbach’s alpha was 0.94, indicating satisfactory reliability.

### 3.3 Control variables

In this study, we included teachers’ seniority and level of kindergarten as control variables because of their underlying, credible effects on the results.

### 3.4 Data analysis

To ensure the objectivity and veracity of the results of the study, the questionnaire was anonymously completed on the Internet. After answering the questions anonymously through the web link, teachers presented the completed scale through the online platform. In this study, IBM SPSS 23.0 was used for data sorting, reliability testing, descriptive statistics, common method variance testing, and correlation analysis. The SPSS PROCESS macro was used to test the mediating effect and moderating effect models (Hayes, 2013).

## 4 Results

### 4.1 Common method deviation test

To avoid common method bias in this study, the Harman single-factor method was used for common method deviation testing (Podsakoff et al., 2003). After principal component analysis, nine eigenvalues greater than 1 were extracted. The first factor explained 33.36% of the variance, which is lower than the 40% required by the critical standard, so this study did not suffer from common method deviation.

### 4.2 Descriptive statistics and correlation analysis

The findings of the mean, standard deviation, skewness, kurtosis, and correlation analysis of each variable are presented in Table 2. According to the criteria set forth by Kline (2005), i.e., the absolute value of skewness is less than three and the absolute value of kurtosis is less than ten, all variables basically obey normal distribution。.

**Table 2:** Descriptive analysis results.

Variables	1	2	3	4
1. Organizational climate	1			
2. Professional learning	0.621***	1		
3. Collective efficacy	0.473***	0.628***	1	
4. Mindfulness in teaching	0.529***	0.373***	0.403***	1
M	0.026	4.443	4.362	3.993
SD	4.016	0.747	0.741	0.578
Skewness	−0.839	−1.565	−1.369	−0.584
Kurtosis	0.498	2.554	1.689	−0.026

*N* = 1,095; ***p < 0.001.

The results of the correlation analysis showed that kindergarten OC was positively related to TPL (r = 0.621, *p* < 0.001), teachers’ CE (r = 0.473, *p* < 0.001), and mindfulness in teaching (r = 0.529, *p* < 0.001). TPL was positively related to teachers’ CE (r = 0.628, *p* < 0.001) and mindfulness in teaching (r = 0.373, *p* < 0.001). Additionally, teachers’ CE was positively related to mindfulness in teaching (r = 0.403, *p* < 0.001).

### 4.3 Moderated mediation model

**Figure 2:**
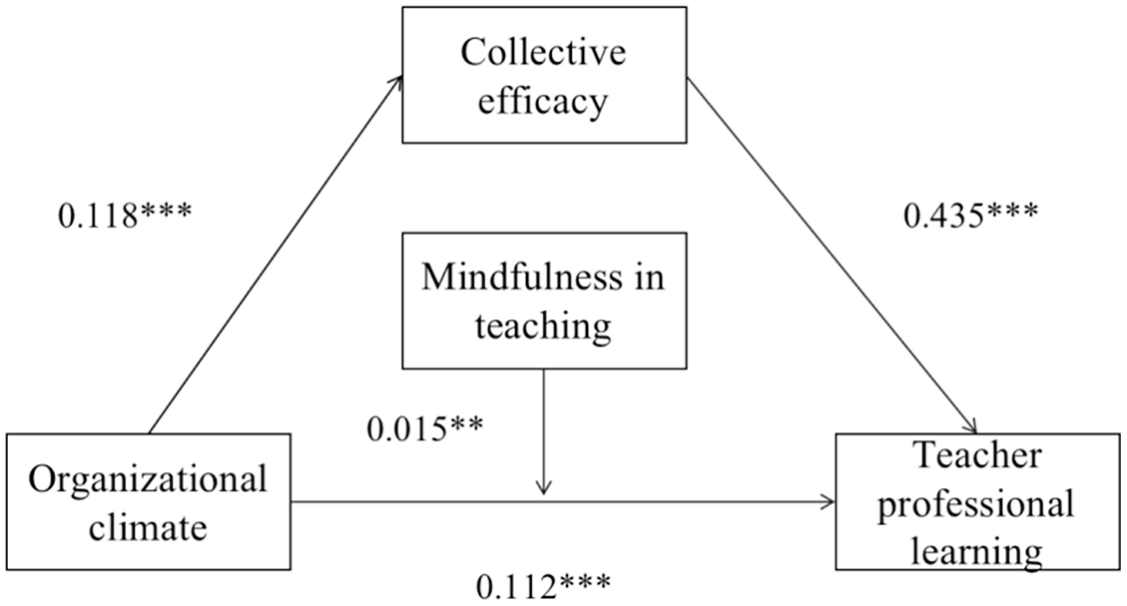
Moderated mediation model; **p < 0.01; ***p < 0.001.

To verify our hypotheses, we used Model 4 in the PROCESS macro to test for mediating effects and Model 5 in the PROCESS macro to test the moderated mediated effects. In this study, all continuous variables were standardized before analysis. The model examined the associations among OC, TPL, CE, and mindfulness in teaching, with teaching experience and kindergarten level as the control variables in all regression models, TPL as the outcome variable, OC as the predictor, CE as the mediating variable, and mindfulness in teaching as the moderating variable, and the findings are given in Table 3 and Figure 2.

**Table 3:** Regression results for conditional indirect effects.

Variables	Outcomes									
	Collective efficacy					Teacher professional learning				
	*β*	SE	*t*	95% CI		*β*	SE	*t*	95%CI	
TS	−0.012	0.023	−0.514	−0.057	0.033	−0.018	0.018	−1.002	−0.053	0.017
KL	0.014	0.037	0.382	−0.059	0.087	0.033	0.029	1.137	−0.024	0.090
OC	0.118	0.007	17.473***	0.105	0.132	0.111	0.007	16.895***	0.098	0.124
MT						−0.009	0.026	−0.343	−0.060	0.042
OC × MT						0.015	0.005	2.729**	0.004	0.025
CE						0.435	0.024	18.120***	0.388	0.482
R	0.473					0.731				
R^2^	0.224					0.535				
F	104.903***					208.308***				

*N* = 1,095. TS, teaching seniority; KL, kindergarten level; OC, organizational climate; MT, mindfulness in teaching; CE, collective efficacy. **p < 0.01; ***p < 0.001.

First, in a mediating-effect test of PROCESS model 4, kindergarten OC positively predicted TPL (*β* = 0.105, *p* < 0.001) and teachers’ CE (*β* = 0.118, *p* < 0.001), and teachers’ CE was positively related to TPL (*β* = 0.430, *p* < 0.001). These results confirm hypothesis H1. The results of further mediating-effect tests showed that teachers’ CE partially mediated the relationship between OC and TPL [SE = 0.006, 95% CI (0.041, 0.061)]. The indirect effect was 0.051 and the total effect was 0.156. In conclusion, the indirect effect accounted for 33% of the total effect. This result suggests that teachers’ CE mediated 33% of the effect of OC on TPL. These results confirm hypothesis H2.

Next, the moderating effect of mindfulness in teaching was validated using PROCESS Model 5. The interaction between OC and MT contributed significantly to TPL (*β* = 0.015, *t* = 2.729, *p* < 0.01). The 95% bootstrap CI was [0.004, 0.025], excluding zero. These results support hypothesis H3. Mindfulness in teaching moderated the effect of OC on TPL.

To probe further the interactions between OC and mindfulness in teaching, conditional regression lines of OC were plotted at one standard deviation below and above the mean for mindfulness in teaching, labeled as low and high mindfulness in teaching in Table 4.

**Table 4:** Conditional effects of organizational climate (OC) moderated by mindfulness in teaching.

Mindfulness in teaching	Effect	SE	Bootstrap lower-limit CI	Bootstrap upper-limit CI
Low (M − 1SD)	0.097	0.008	0.081	0.112
Mean (M)	0.111	0.007	0.098	0.124
High (M + 1SD)	0.126	0.009	0.108	0.144

**Figure 3:**
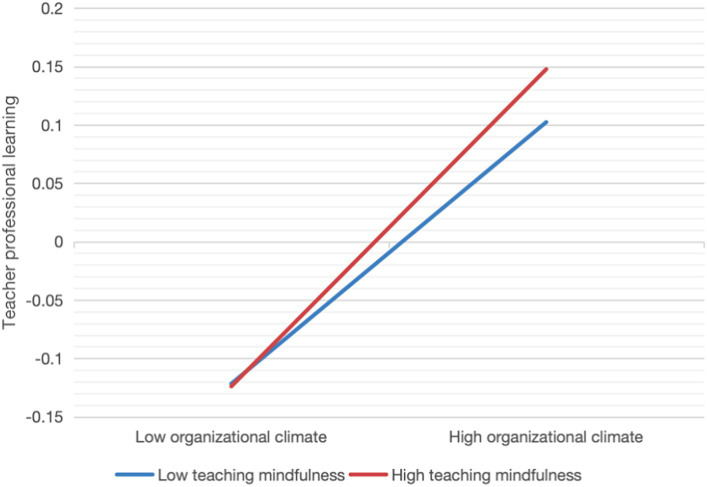
Interaction effect of mindfulness in teaching and OC in predicting teacher professional learning.

Regardless of the level of mindfulness in teaching, OC is always positively related to TPL with a stronger relationship. Additionally, we explain this significant interaction via a simple slope (Figure 3). The results show that kindergarten OC was a significant positive predictor of TPL in the high positive group, and in the low positive group, kindergarten OC was also a significant positive predictor of TPL, but the prediction was stronger in the high positive group compared to the latter. In addition, at the same OC level, high mindfulness in teaching might have higher TPL levels than low mindfulness in teaching, supporting hypothesis H3.

## 5 Discussion

In this study, we explored the role of kindergarten OC in influencing TPL, and based on the job demands–resources (JDR) model and related literature on TPL, we proposed a moderated mediation model. The results showed that as a critical external resource for TPL, kindergarten OC can positively influence teachers in their daily educational practices and promote their professional learning by enhancing their collective sense of efficacy. In addition, as an internal resource for individual teachers, mindfulness in teaching plays a moderating role in this process. The findings of this research offer empirical support for the pathway of kindergarten OC influencing TPL, and they highlight the mediating role of teachers’ CE and the moderating role of mindfulness in teaching. This leads to further in-depth insights into the influencing mechanisms of TPL and provides a reliable and feasible basis for promoting TPL.

### 5.1 Theoretical implications

This study has several theoretical implications. First, it was focused on how TPL is influenced by the OC of kindergartens, thereby enriching the literature on OC and TPL by expanding on the specific application of kindergarten OC to TPL. Past studies on TPL have been focused on the perspective of individual teachers or the influence of leader traits and styles but have neglected how influential the role of climate is provided by the organization as a whole (Bektaş et al., 2022). Previous related research has identified school conditions as a significant predictor of TPL (Opfer and Pedder, 2011; Admiraal et al., 2016; Huang et al., 2020), but previous research neglected the interaction and collaboration among colleagues in the OC.

First, this paper expands the perspective to the level of OC as a holistic organizational characteristic, taking into account not only the supervisory, supportive, and leading roles of leaders but also the interactive factors among teachers, to comprehensively examine the impact of organizational-level climate conditions on TPL, responding to the previous discussion on the significant relationship between school professional climate and teachers’ participation in learning activities (Patrick, 2022). The findings show that TPL is indeed influenced by the overall OC, which strongly suggests that it is particularly important to view the kindergarten as a whole and to create a holistic and harmonious interpersonal climate (rather than just a single interactive perspective of a leader and teachers). Similar results can be found in previous research, where a supportive organizational environment and organizational culture can significantly influence individual formal and informal learning (Jurasaite-Harbison and Rex, 2010; Choi and Jacobs, 2011; Huang and Wang, 2021). Early childhood educators perform better in environments where they have trust in their colleagues and leaders (Yu and Chen, 2023). As one of the work resources of teachers, OC can help them understand their professional identity and enhance their role beliefs (Andreasen et al., 2019). Teachers’ perceived school climate significantly influences their cognitive development, attitudinal feelings, and behavioral performance (Dicke et al., 2015; Malinen and Savolainen, 2016).

Second, from the perspective of perceptions of efficacy, this article interprets the deeper mechanisms by which the OC of kindergartens influences TPL and corroborates the mediating role played by CE. Both social cognitive theory and the JDR model emphasize that support from the school can act on teachers’ behavior and performance through their perceptions of efficacy (Bandura, 1977; Xanthopoulou et al., 2007). This study found that the OC of kindergartens had an indirect effect on TPL through the teachers’ collective sense of efficacy. That is, an open and positive OC inspires teachers’ perceptions of collective potential and enhances teachers’ beliefs about kindergarten competence, which will further motivate TPL. This is in line with previous research findings that a supportive OC in schools characterized by reflective dialog can strengthen teachers’ sense of CE (Lim and Eo, 2014; Zhang et al., 2023). When teachers have a high sense of CE, they are more likely to accept challenging goals and act persistently, which leads to better performance (Goddard et al., 2000). Put another way, teachers who feel a supportive, collaborative, and intimate atmosphere in their organizations are more likely to feel collectively empowered and more confident in collaborating with colleagues and in their learning and development.

Finally, based on the JDR model, the work environment conditions related to TPL activities can be divided into two groups: job demands and work resources (Bakker and Demerouti, 2017). Teacher learning will take place in work settings with more or higher work resources. Yet, the JDR model ignores the mental mechanisms that account for the positive impact of job resources on teacher learning (Schaufeli and Taris, 2014). The awareness of these mental mechanisms is significant as it offers a new explanatory perspective on why there are still differences in teachers’ professional learning outcomes under the same working conditions. The findings of this research confirm that the psychological mechanism of teachers’ mindfulness in teaching—as a personal resource for teachers—plays a moderating role in the relationship between the OC of kindergartens and TPL. Specifically, when kindergarten teachers have a higher level of mindfulness in teaching, they are more likely to perceive a positive and open climate that further facilitates their professional learning. Conversely, when teachers have low levels of mindfulness, it is difficult for them to perceive a positive OC, and the process of professional learning becomes difficult. Therefore, it can be suggested that teachers’ higher levels of mindfulness in teaching strengthens the positive effect of kindergarten OC on professional learning. This is consistent with prior studies showing that the engagement of mindfulness supports professionals’ acceptance, positivity, and responsiveness to others, and positively influences teacher job engagement and teacher well-being (Malinowski and Lim, 2015; DeMauro et al., 2019). The risk buffer model suggests that protective elements can cushion or diminish the negative impacts of risk factors (Fergus and Zimmerman, 2005).

Therefore, we can infer that the protective factors not only diminish the effect of unfavorable factors but also buffer and promote the conversion of favorable factors. Prior research has also suggested that mindfulness not only weakens the negative impact of overwork on the teacher–child relationship but also has a significant buffering and protective effect on kindergarten teachers’ perceptions of the work environment on the quality of the teacher-child relationship (Yang et al., 2023). Higher levels of mindfulness in teaching can help teachers focus on the present moment, and teachers no longer need additional energy to cope with adverse external circumstances or negative internal feelings and emotions in their classroom teaching, management, and relationships with children (Emerson et al., 2017). It follows that mindfulness can have a significant and positive impact on ECEC. Research has shown that teacher mindfulness can be enhanced and changed through training, which can help teachers to engage in classroom instruction and professional learning from cognitive, emotional, and experiential perspectives (Yuan et al., 2023). Thus, our study also reaffirms the difference in the role of personal resources in external resources and personal development.

### 5.2 Practical implications

The professional learning of kindergarten teachers is the key to the development of high-quality ECEC, but from a practical point of view, there is not enough research to explore the specific impact of the climate of kindergarten as an organization, as a whole, on teachers’ professional learning. Therefore, this study examines the impact of kindergarten OC on TPL from this perspective, providing new insights for kindergarten administrators and teachers.

It can be inferred from the findings of this research that TPL can be motivated in various ways, including an open OC, CE, and positive personal psychological adjustment. First of all, this study suggests that an open OC can facilitate the effectiveness of TPL. In creating a positive and open OC, it is important to focus on not only the supportive and supervisory roles of leaders but also the cooperative communication and emotional interactions among teachers that play an essential role in their professional learning. Therefore, in addition to focusing on their behaviors and attitudes in the overall organizational culture and climate, kindergarten leaders and related managers should also motivate teachers to consult with each other, study and discuss together, and focus on creating an open, positive, and supportive OC.

Second, the OC of kindergartens contributes to teachers’ perceptions of kindergarten potential and plays a direct or indirect role in the process of TPL. As mentioned in some studies, there is a coupling effect between teachers’ CE—as a powerful force for learning among and within schools—and TPL (Loughland and Ryan, 2022). The results of this study demonstrate that teachers’ CE can be developed in team building where TPL is an important endeavor. This is because learning teams provide opportunities for teachers to learn indirectly from the experiences of colleagues and provide emotional support for each other, which gives them a deep sense of trust and confidence in the organization of which they are a part. Therefore, it is necessary to create the right environmental conditions for the motivational structure of teachers’ CE, which includes effective behaviors of leaders and effective interactions among colleagues based on the improvement of teachers’ CE.

Finally, from an intra-individual resource perspective, the feelings, emotions, and attitudes of kindergarten teachers in the present moment must have a positive effect on their professional learning. In addition, promoting mindfulness in teaching is a useful intervention to prevent kindergarten teachers from being overworked (Ma et al., 2021). Incorporating mindfulness into ECEC settings serves as a mechanism to transform the individual teacher’s personal connections to stress and improve their well-being (Hatton-Bowers et al., 2022), and improve the ability of ECEC professionals to be present, conscious, and happier with their children (Hatton-Bowers et al., 2020). For kindergarten teachers, using mindfulness to reduce their stress and increase their well-being in educational settings can increase teachers’ engagement in educational practice and have a beneficial impact on their professional development process (Roeser et al., 2012). Therefore, kindergarten leaders and teacher education researchers need to focus on consciously and consistently training teachers in teaching mindfulness, and ECEC professionals themselves can put into practice mindfulness-in-teaching modules in their professional practice to ensure efficient use of external resources for self-development.

### 5.3 Limitations and future research directions

This research certainly has some limitations. First, we adopted a cross-sectional data collection approach and did not collect data separately from multiple periods, so the explanatory strength of causal interpretations among variables was weak. In the future, multiple time-point follow-up surveys could be used for in-depth investigation and validation to enhance the explanatory power of causal inferences among variables. Second, the data in this study were all surveyed using teacher self-reports, focusing on individual perspectives of teachers, and although no serious common method deviation problems were found in the study, the relationships among variables may be exaggerated as a result. In the future, we could consider multi-agent and multi-method evaluation of organizational atmosphere (such as using the evaluation of the principal or conducting observation analysis) to enhance the objectivity of the research. Finally, the focus of this study was on the mediating role of teachers’ CE and the moderating role of individual traits, but TPL is a process of interaction with groups and organizations. In the process of OC influencing TPL, peer relationships and conditions of the organizational context will influence the mechanisms of action of this process. Future studies could examine the relationship among elements of external conditions related to TPL, such as interactions and learning conditions provided in kindergartens, and OC and TPL, and further discuss the mechanisms at play.

## Data availability statement

The raw data supporting the conclusions of this article will be made available by the authors, without undue reservation.

## Ethics statement

The studies involving human participants were reviewed and approved by Institutional Review Board of Northeast Normal University. The studies were conducted in accordance with the local legislation and institutional requirements. The participants provided their written informed consent to participate in this study.

## Author contributions

SH: Conceptualization, Data curation, Formal analysis, Investigation, Methodology, Software, Writing – original draft, Writing – review & editing. DY: Investigation, Resources, Supervision, Visualization, Writing – review & editing. LF: Conceptualization, Data curation, Investigation, Methodology, Writing – original draft.
